# Correction: [(3-Nitro-1*H*-1,2,4-triazol-1-yl)-*NNO*-azoxy]furazans: energetic materials containing an N(O)=N–N fragment

**DOI:** 10.1039/d1ra90134f

**Published:** 2021-07-29

**Authors:** Dmitry A. Gulyaev, Michael S. Klenov, Aleksandr M. Churakov, Yurii A. Strelenko, Ivan V. Fedyanin, David B. Lempert, Ekaterina K. Kosareva, Tatiana S. Kon’kova, Yurii N. Matyushin, Vladimir A. Tartakovsky

**Affiliations:** N. D. Zelinsky Institute of Organic Chemistry, Russian Academy of Sciences Moscow 119991 Russian Federation klenov@ioc.ac.ru churakov@ioc.ac.ru; A. N. Nesmeyanov Institute of Organoelement Compounds, Russian Academy of Sciences Moscow 119991 Russian Federation; Plekhanov Russian University of Economics Moscow 117997 Russian Federation; Institute of Problems of Chemical Physics, Russian Academy of Sciences Chernogolovka Moscow region 142432 Russian Federation; N. N. Semenov Federal Research Center for Chemical Physics, Russian Academy of Sciences Moscow 119991 Russian Federation

## Abstract

Correction for ‘[(3-Nitro-1*H*-1,2,4-triazol-1-yl)-*NNO*-azoxy]furazans: energetic materials containing an N(O)=N–N fragment’ by Dmitry A. Gulyaev *et al.*, *RSC Adv.*, 2021, **11**, 24013–24021, DOI: 10.1039/D1RA03919A.

In the original article, in Table 2 the formula “
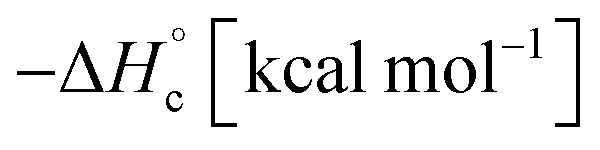
^*i*^” was shown incorrectly. The corrected formula should read as “
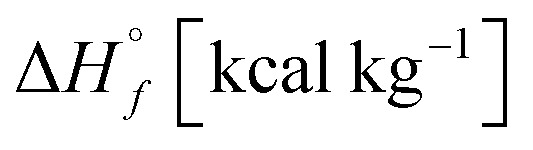
^*i*^”.

The Royal Society of Chemistry apologises for these errors and any consequent inconvenience to authors and readers.

## Supplementary Material

